# Penile and scrotal strangulation caused by a steel ring: a case report

**DOI:** 10.1186/1757-1626-1-45

**Published:** 2008-07-18

**Authors:** Ioannis Efthimiou, Savas Kazoulis, Ioannis Christoulakis

**Affiliations:** 1 General Hospital of Chania "Agios Georgios", Chania, Crete, TK73100, Greece

## Abstract

Application of constricting devices on the external male genitalia for increasing sexual performance is an unusual practice that can potentially lead to penile strangulation with severe consequences. In this case report we describe a case of a 48 year old male who presented in our hospital with a steel ring on his external genitalia which led to penile strangulation and a short review of the literature. The foreign body was successfully removed by an angle grinder which was not immediately available in the operating theatre. The patient had an uneventful recovery.

## Introduction

Entrapment of metal rings that strangulate the shaft of the penis is a rare emergency in urology. Removal of these may be challenging for the Urologist [1]. If the condition is left untreated there are potentially dangerous consequences for the patient. In this case report we describe a case report of a 48 year old male who presented in our hospital with a steel ring on his external genitalia which led to penile strangulation and was removed by an angle grinder which was not immediately available in the operating theatre with a short review of the literature.

## Case presentation and discussion

**Figure 1:**
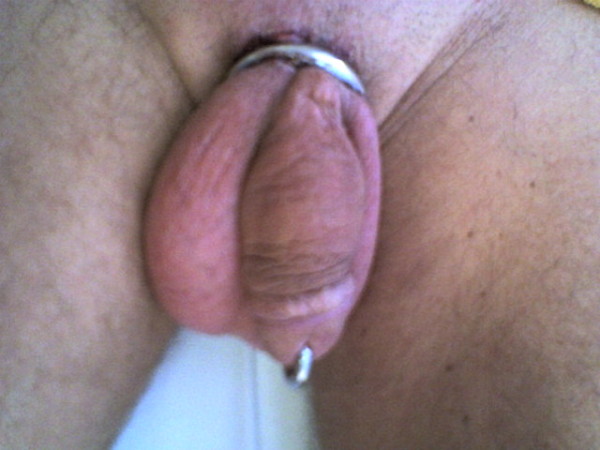
Before removal of the steel ring. It is obvious the local oedema.

**Figure 2:**
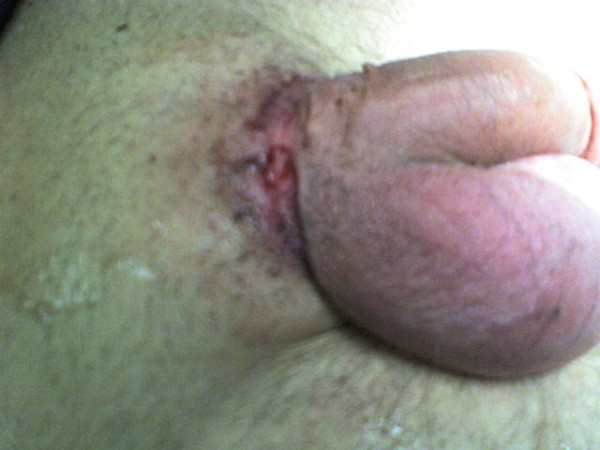
After removal of the steel ring. There is ulcer necrosis of the skin on the pressure points.

A 48 year old Englishman presented in our hospital with a steel ring constricting his external genitalia that he could not remove. The ring had been placed for enhancement of sexual performance 48 hours ago. He complained of pain and swelling on his external genitalia but he did not report any difficulty in passing urine. On clinical examination he had normal vital signs and there was marked local oedema with ulceration and pus at the pressure points (figure 1). Attempts to remove it with lubrication, compression or cutting devices from the department of orthopaedics were unsuccessful. Further attempts to cut it with the biggest bold cutters that were available in the market failed again. The patient was taken to the operating theatre and under general anaesthesia the ring was cut along two sides with the help of an angle grinder that was supplied by our hospital's department of engineering (figure 2). To avoid burns from the sparks and excessive heating, the penis was isolated with pieces of tinfoil between the ring and the skin and pouring cold normal saline on the field. The ring was successfully removed and the patient was started on intravenous antibiotics. 24 hours later the oedema had subsided and the patient was discharged.

A great variety of metallic and non-metallic rings causing constriction the external genitalia has been described in literature [1]. The type of the foreign body differs in relation to age. In newborns and children penile strangulation with air or elastic rings has been described. The insertion of these rings may be accidental or intentional from the patient, a sibling or the parent [2,3]. In adults various objects like wedding rings, metal plumbing cuffs, bull rings, bottle necks etc have been used [1]. In adolescents it is usually the result of curiosity or masturbation while in adults the reason is to enhance sexual pleasure, autoerotism or as a result of a psychiatric disorder.

Insertion of constricting rings in the flaccid or semi erect penis may result to inability in removing them after erection. The object has been placed a few hours up to 3–4 days before seeking medical help and the patients have usually attempted unsuccessfully to remove the object themselves [1,4].

In children the strangulation may be easily overlooked as an erosion or eczema. A high level of suspicion is required from the clinician to avoid missing it.

Within a few hours of obstruction of the blood supply, as stagnation of blood becomes more prominent, oedema and haemorrhage occur, causing a swelling of the affected part. Metallic rings usually cause less injury than non-metallic rings [5]. Delayed removal may lead to necrosis, fistula, sepsis and penile amputation [4].

Various procedures depending on the constricting object have been described for removal. These include the common metal ring cutter, cutting tang, metal saw, Dremel Moto-Tool Kit, Anspach cement eater, high speed drill, string method, and "wrapping" by package cord [1].

Usually removal is accomplished under general anaesthesia. The urologist must remove the ring with great care to avoid iatrogenic injury to the external genitalia. It should also be kept in mind that removal of these objects may be challenging and require equipment that is not directly available in the urology department.

## Conclusion

Strangulating foreign bodies of male external genitalia may require resourcefulness in order to be removed safely with no consequences for the patient. General anesthesia may be necessary to facilitate extraction.

## Consent

Written informed consent was obtained from the patient for publication of this case report and accompanying images. A copy of the written consent is available for review by the Editor-in-Chief of this journal.

## Competing interests

The authors declare that they have no competing interests.

## Authors' contributions

EI participated in the operating theatre, prepared and submitted the manuscript and photographs. KS participated in the operating theatre and collected the patient data. CI revised the manuscript. All authors read and approved the final manuscript.
